# Photocatalytic nucleophilic addition of alcohols to styrenes in Markovnikov and anti-Markovnikov orientation

**DOI:** 10.3762/bjoc.11.62

**Published:** 2015-04-27

**Authors:** Martin Weiser, Sergej Hermann, Alexander Penner, Hans-Achim Wagenknecht

**Affiliations:** 1 Institute of Organic Chemistry, Karlsruhe Institute of Technology (KIT), Fritz-Haber-Weg 6, 76131 Karlsruhe, Germany

**Keywords:** electron transfer, perylene bisimide, photocatalysis, photochemistry, pyrene

## Abstract

The nucleophilic addition of methanol and other alcohols to 1,1-diphenylethylene (**1**) and styrene (**6**) into the Markovnikov- and anti-Markovnikov-type products was selectively achieved with 1-(*N*,*N*-dimethylamino)pyrene (Py) and 1,7-dicyanoperylene-3,4:9,10-tetracarboxylic acid bisimide (PDI) as photoredox catalysts. The regioselectivity was controlled by the photocatalyst. For the reductive mode towards the Markovnikov-type regioselectivity, Py was applied as photocatalyst and triethylamine as electron shuttle. This approach was also used for intramolecular additions. For the oxidative mode towards the anti-Markovnikov-type regioselectivety, PDI was applied together with Ph–SH as additive. Photocatalytic additions of a variety of alcohols gave the corresponding products in good to excellent yields. The proposed photocatalytic electron transfer mechanism was supported by detection of the PDI radical anion as key intermediate and by comparison of two intramolecular reactions with different electron density. Representative mesoflow reactor experiments allowed to significantly shorten the irradiation times and to use sunlight as “green” light source.

## Introduction

Photocatalysts are organic or inorganic compounds that couple the physical process of light absorption with a chemical reaction by means of time, space and energetics, in order to catalyse it. With respect to the “green” character of sunlight as unlimited natural light source and the availability of LEDs as cheap and reliable artificial light sources, the research field of photoredox catalysis has tremendously grown over the past decade [1–7]. Transition metal complexes, mainly [Ru(bpy)_3_]^2+^ [7], were most often used as photocatalysts, whereas the potential of organic compounds and dyes has not yet been fully exploited [8]. The way towards a really complete organo-type photoredox catalysis has mainly been established for eosin Y as an important alternative for [Ru(bpy)_3_]^2+^ [9].

Photocatalytic nucleophilic additions of amines and alcohols to olefins, especially styrenes, became an increasingly important task due to their potential and versatile applicability in chemical syntheses. Their non-photochemical counterparts require acids, bases or transition metal complexes as catalysts [10]. The first examples of photochemical olefin aminations were reported by Cookson et al. [11] and Kawanisi et al. [12] in the 1960s/70s, and Lewis identified exciplex states as key intermediates [13–14]. The corresponding photohydration worked only if the aromatic olefins as starting material were directly excited by UV light [15–16]. The first approach towards a photocatalytic version of this type of reaction came from Arnold, Maroulis et al. [17–18]. They demonstrated that electron-rich naphthalenes are able to photoinitiate methanol additions to olefins into the Markovnikov orientation and proposed an oxidative electron transfer mechanism for this process [17]. Complementarily, electron-poor naphthalenes yielded the anti-Markovnikov-type addition of cyanide to styrene [18]. Recently, we showed by a library of different chromophores that 1-(*N*,*N*-dimethylamino)pyrene (Py) can be applied as photocatalyst for the nucleophilic addition of methanol to styrene derivatives into the Markovnikov orientation [19]. Most recently, Nicewicz et al. published the hydrofunctionalization of alkenes to the anti-Markovnikov products by photoredox catalysis using 9-mesityl-10-methylacridinium [20–21]. Herein, we want to present our complementary approach to perform inter- and intramolecular nucleophilic additions of alcohols to styrene derivatives by photocatalysis. The regioselectivity – Markovnikov or anti-Markovnikov – can simply be controlled by the chosen photocatalyst, either Py or 1,7-dicyanoperylene-3,4:9,10-tetracarboxylic acid bisimide (PDI).

## Results and Discussion

### Photocatalytic complementarity

**Scheme 1:**
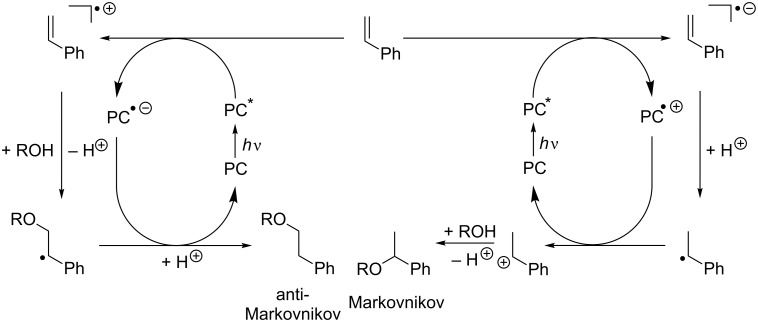
Photooxidation of the substrate and reductive quenching of the photocatalyst (left) vs photoreduction of the substrate and oxidative quenching of the photocatalyst (right) give two complementary photocatalytic cycles yielding either anti-Markovnikov-type or Markovnikov-type selectivity for the nucleophilic addition of alcohols to styrene derivatives.

The photocatalytic complementarity of the two different routes (to the Markovnikov or anti-Markovnikov addition products of styrene derivatives) results from the two types of photoinduced charge transfer initiated by the photoexcited catalyst (Scheme 1). If an electron-poor chromophore is applied, the first step that follows irradiation is an electron transfer leading to one-electron oxidation of the substrate styrene and may involve intermediates such as exciplexes. Nucleophilic attack and loss of the proton of the alcohol yield a radical at the benzylic position that explains the anti-Markovnikov-type selectivity of this photocatalytic process. Back charge transfer to the photocatalyst closes the photocatalytic cycle and subsequent protonation yields the anti-Markovnikov-type addition product. In contrast, an electron-rich chromophore photoinduces an electron transfer onto the substrate. The corresponding radical anion is protonated rapidly to the neutral radical that is the key intermediate to explain the Markovnikov selectivity of this route. Both steps, electron transfer and protonation, could also occur in one proton-coupled electron transfer step. Back electron transfer to the photocatalyst finishes the photocatalytic cycle of this process, and subsequent nucleophilic attack accompanied by deprotonation gives the Markovnikov-type addition product.

### Reductive route: Markovnikov regioselectivity

**Scheme 2:**
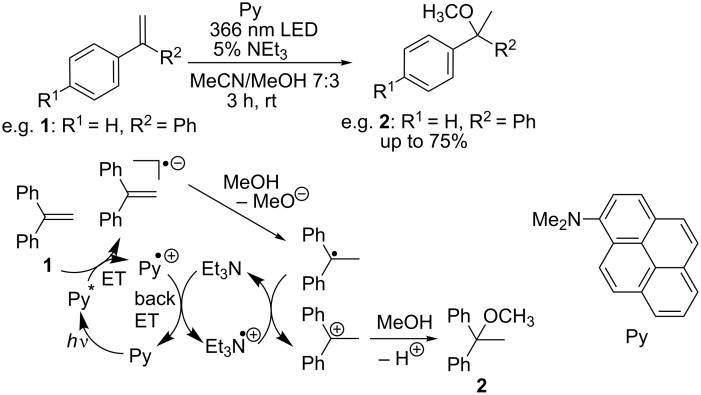
Mechanism of the Markovnikov-type photocatalytic addition of methanol to 1,1-diphenylethylene (**1**) and other α-phenylstyrenes in the presence of 10 mol % of Py (ET = electron transfer).

For the reductive mode of photocatalysis towards the Markovnikov-oriented addition products, we recently applied Py as photocatalyst and 1,1-diphenylethylene (**1**) as test substrate (Scheme 2). It was assumed that inefficient back electron transfer was responsible for low yields of the MeOH addition product **2** and rapid degradation of the photocatalyst Py. This problem could be solved by adding triethylamine which served as electron shuttle between back electron transfer that regenerates the photocatalyst and the final step of product formation. The substrate scope of this optimized photocatalytic conditions revealed that electron-poor α-phenylstyrenes and styrenes are preferred which further supported the reductive electron transfer mechanism [19].

**Scheme 3:**
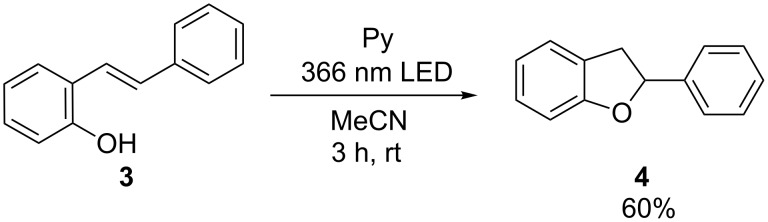
Intramolecular photocatalytic addition with substrate **3**; reaction conditions: **3** (2 mM), Py (2 mM), in MeCN, argon atmosphere, 3 h, 25 °C, 366 nm high-power LED, **3** and **4** identified and quantified by GC–MS.

This photocatalysis was applied also for intramolecular additions. In the particular case of substrate **3**, Et_3_N as electron shuttle could not be used; it provided a competing nucleophile since the desired nucleophile could not be added in high excess. In order to shift the reaction more towards the intramolecular alternative, the photoredox catalysis was performed in high dilution (2 mM). The product **4** could be identified in 60% yield (Scheme 3).

This example showed that the addition of Et_3_N as electron shuttle was not required in all cases. A more detailed look on the problem of inefficient back electron transfer indicated that loss of polar attraction after rapid protonation of the substrate radical anion might lead to diffusion and separation of the photocatalyst from the intermediate product-forming radical cation. If it was assumed that back electron transfer was a strongly distance dependant process, the photocatalyst might not be regenerated and hence removed from the catalytic cycle. This scenario could potentially be improved by a substrate binding site on the photocatalyst that keeps the substrate in the vicinity of Py as long as it is required for forward and back electron transfer.

### Oxidative route: anti-Markovnikov regioselectivity

**Scheme 4:**
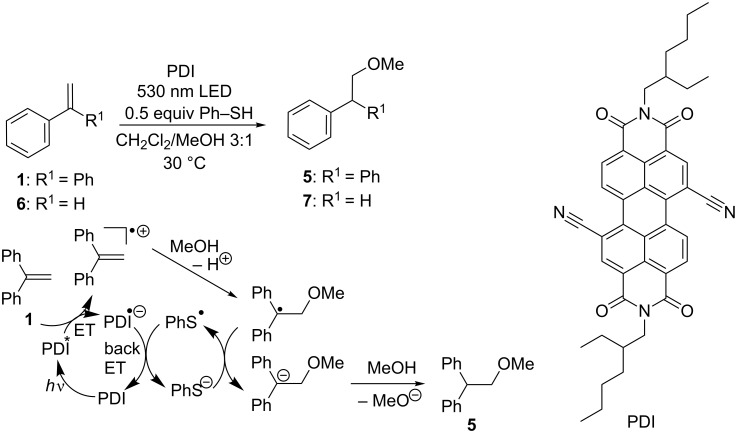
Proposed mechanism of the anti-Markovnikov-type photocatalytic addition of methanol to 1,1-diphenylethylene (**1**) and styrene (**6**) yielding the corresponding products **5** and **7** (ET = electron transfer).

For the oxidative mode of photocatalysis towards the anti-Markovnikov-oriented addition products, PDI (Scheme 4) was applied as photocatalyst. Its absorption maximum in CH_2_Cl_2_ is located at 525 nm that makes it an excellent candidate for photoirradiation by both sunlight and green light-emitting diodes. Furthermore, based on *E*_red_(PDI/PDI^●−^) = −0.28 V (measured by cyclic voltammetry, vs SCE, see Supporting Information File 1) and *E*_00_ = 2.35 eV (see Supporting Information File 1), PDI is an electron deficient chromophore with an excited state oxidation potential of 2.07 V. In combination with the oxidation potential of 1.81 V (vs SCE) [22] for substrate **1** the driving force Δ*G* of initial oxidation was estimated by Rehm–Weller to be around 250 meV. In general, irradiations were carried out in quartz glass cuvettes at a constant temperature of 30 °C, using a 250 mW high-power LED (λ = 530 nm) as light source while stirring.

**Figure 1:**
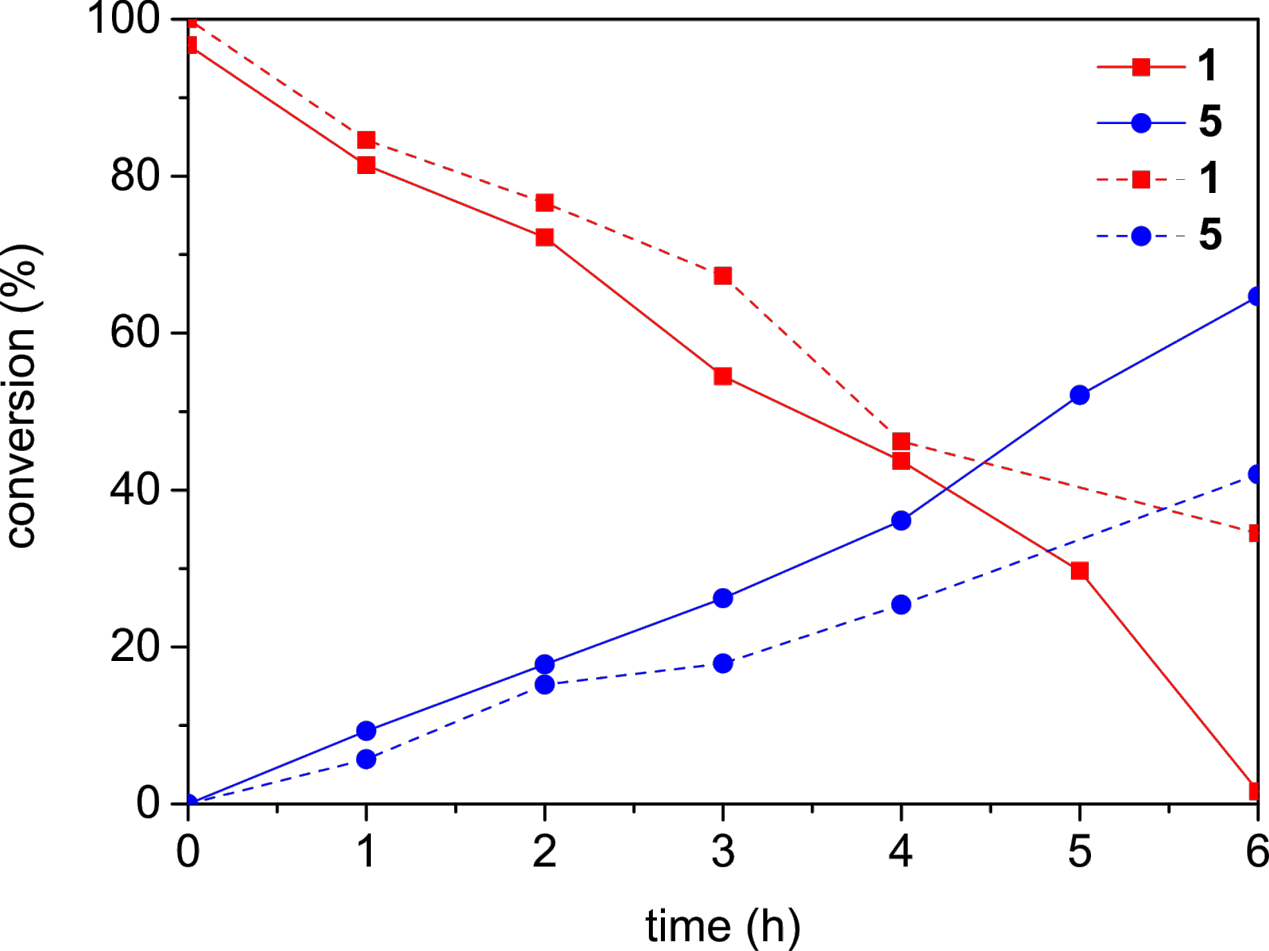
Conversion of substrate **1** and formation of product **5** observed during photocatalysis with PDI in the presence of 0.4 equiv (dashed lines) and 1.0 equiv (solid lines) of Ph–SH as additive; reaction conditions: **1** (20 mM), Ph–SH (20 mM), PDI (0.5 mM), in CH_2_Cl_2_/MeOH 3:1 (4 mL), argon atmosphere, 30 °C, 250 mW LED, λ = 530 nm, **1** and **5** identified and quantified by GC–MS.

Preliminary experiments with substrate **1** revealed that formation of benzophenone was nearly completely prevented by carefully degassing the reaction mixture. A previous report of Neunteufel and Arnold considered the electron transfer from the catalyst onto the substrate as key step [23]. In agreement with that proposal, the Stern–Volmer plots (see Supporting Information File 1) showed that fluorescence of PDI is significantly quenched in the presence of **1**. The critical step, however, seemed to be the back electron transfer that recovers the photocatalyst from the PDI radical anion after nucleophilic addition, since addition of Ph–SH as electron and proton shuttle helped to significantly accelerated reactions [20–21]. In this respect, oxidative and reductive mode behaved similarily since both types of photocatalysis needed a suitable electron shuttle as additive. Comparison of MeOH addition reactions to substrate **1** in the presence of 0.4 and 1.0 equivalents of Ph–SH as additive showed differences in conversion rates, especially during the first six hours of irradiation (Figure 1). With stoichiometric amounts of Ph–SH full conversion was achieved within six hours, whereas 40 mol % only reached 70% of conversion at that time.

Nucleophilic addition of a variety of alcohols to substrate **1** gave the corresponding products in excellent yields (Table 1). Especially the conversion of **1** with benzyl alcohol was significantly slower, since longer irradiations were needed. Only the addition of phenol failed completely. Since isopropanol and *tert*-butanol as sterically demanding nucleophiles gave the corresponding addition products in good yields, it was assumed that the acidity of benzyl alcohol, and more significantly of phenol, weakened the nucleophilicity for this type of reaction. Styrene (**6**) has an oxidation potential of 1.94 V (vs SCE) [22] and, hence, could also be oxidized by the chosen photocatalyst PDI. The corresponding photocatalytic nucleophilic additions to **6** (Table 1) yielded less of each product, which was in agreement with the higher oxidation potential (compared to **1**). Here again, the addition of phenol showed no significant amounts of product formation.

**Table 1:** Photocatalytic nucleophilic addition of alcohols to **1** and **6**^a^.

nucleophile	substrate **1**		substrate **6**
	yields (%)^b^ of **5** after 12 h irradiation	yields (%)^b^ of **5** after 24 h irradiation	yields (%)^b^ of **7** after 42 h irradiation

methanol	69	100	32
ethanol	75	100	21
propanol	64	100	24
butanol	66	100	21
isopropanol	63	100	19
*tert*-butanol	78	98	19
benzyl alcohol	52	84	8^c^
phenol	n.d.	0	0

^a^Reaction conditions: **1** or **6** (25 mM), Ph–SH (12.5 mM), PDI (0.5 mM), in CH_2_Cl_2_/alcohol 3:1 (4 mL), argon atmosphere, 30 °C, 250 mW LED, λ = 530 nm, **1**, **6** and products identified and quantified by GC–MS. ^b^Averaged yield from at least two independent reactions. As no byproducts have been detected conversion matches yield. ^c^Conversion = 24%.

**Figure 2:**
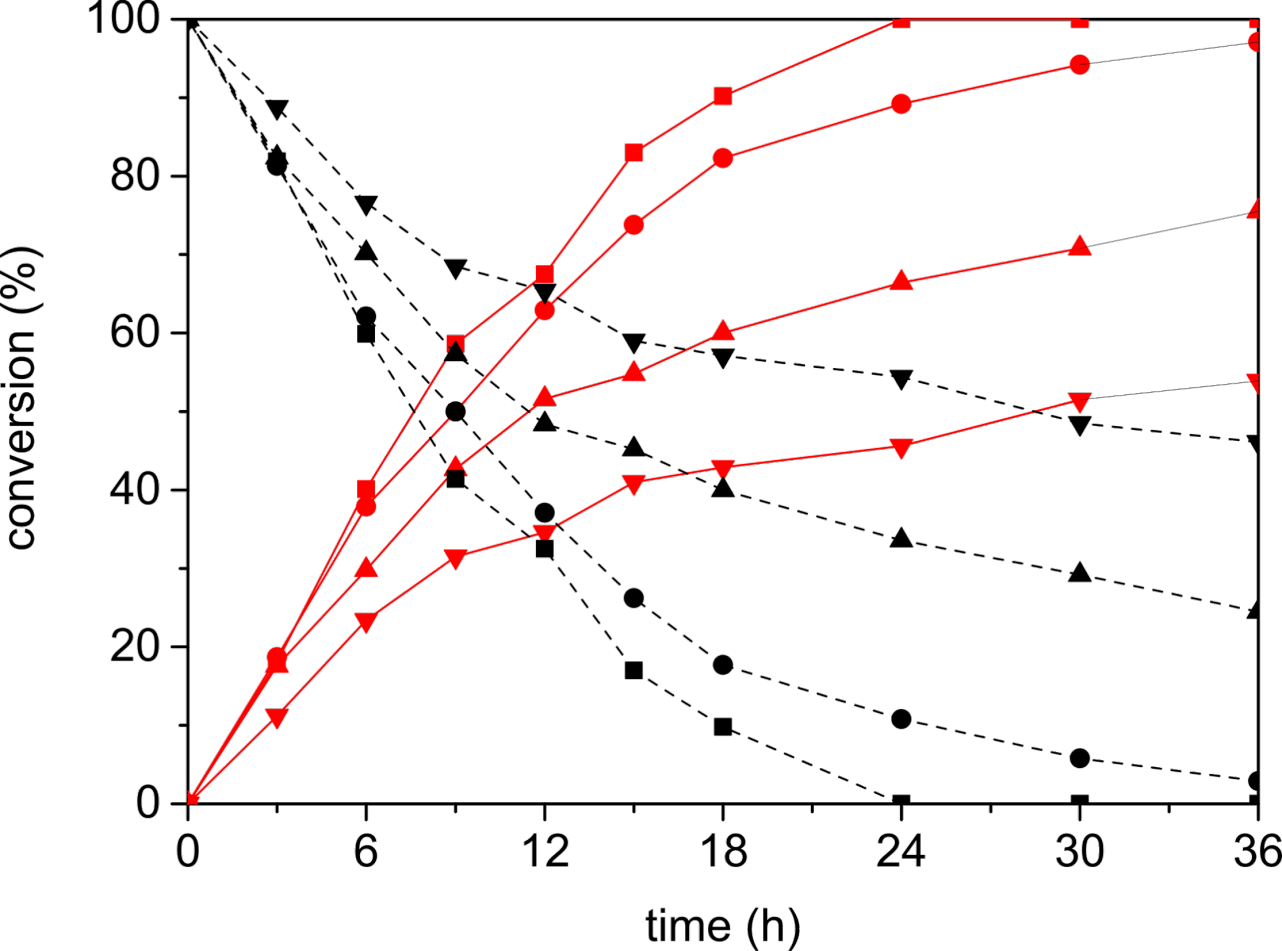
Conversion of substrate **1** (black dashed) and formation of product **5** (red solid) observed during photocatalysis with different amounts of PDI as photocatalyst; reaction conditions: **1** (25 mM), Ph–SH (12.5 mM), PDI (0.05 (▼), 0.125 (▲), 0.25 (●), 0.50 (■) mM), in CH_2_Cl_2_/alcohol 3:1 (4 mL), argon atmosphere, 30 °C, 250 mW LED, λ = 530 nm, **1** and **5** identified and quantified by GC–MS.

We representatively demonstrated the dependency of the performance of photocatalysis with substrate **1** on different PDI concentrations (Figure 2). After three hours, the yields of methanol addition product **5** differed only slightly, but on a longer timescale (12 h and longer) the yields diverged as expected. The reaction with 2 mol % of PDI was finished after 24 h, whereas the reaction with only 1 mol % reached full conversion only after 12 additional hours of irradiation time. The usage of just 0.2 and 0.5 mol % PDI increased the irradiation time at least to 36 h, and it was considered doubtful if prolonged irradiation would complete the reactions.

**Figure 3:**
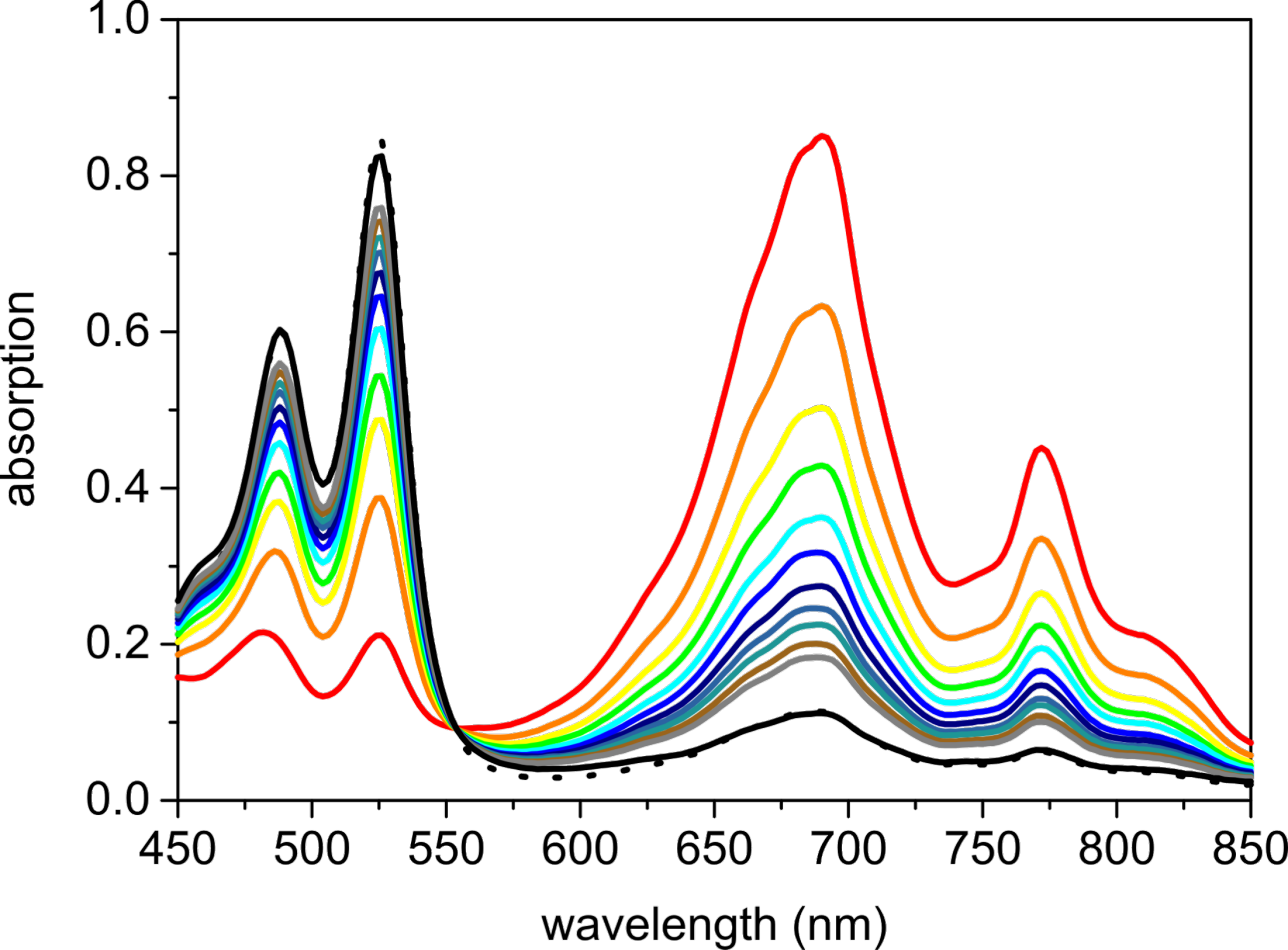
Spectra of PDI before (dotted black) and after excitation (red), then every 2 min until ground state is reached after 30 min (solid black). Reaction conditions: **1** (25 mM), Ph–SH (12.5 mM), PDI (0.02 mM), in CH_2_Cl_2_/MeOH 3:1 (4 mL), argon atmosphere, 25 °C, irradiation by 2 LEDs (250 mW), λ = 530 nm.

During these photocatalytic experiments, the colour of the solution changed from orange to blue after the first seconds of irradiation and turned back to orange just when the reaction was finished. If the irradiation of the photocatalytic sample was stopped it took about an hour until the blue color completely disappeared and obviously the chromophore relaxed back to the ground state. Spectroelectrochemistry measurements (see Supporting Information File 1) revealed that the blue colored intermediate could be assigned to the radical anion of PDI as photocatalyst whose half-lifetime was determined to be approximately 4 min (Figure 3). The appearance of this intermediate strongly supported the proposed electron transfer mechanism of this type of photocatalysis (Scheme 4).

**Scheme 5:**
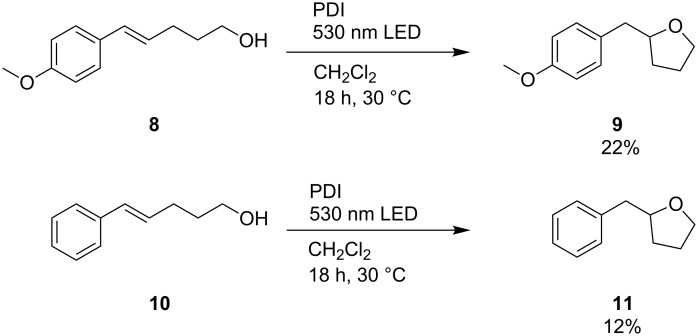
Intramolecular additions of substrates **8** and **10** to demonstrate the effect of different electron densities of the double bond. Reaction conditions: **8** or **10** (25 mM), Ph–SH (12.5 mM), PDI (0.5 mM), in CH_2_Cl_2_ (4 mL), argon atmosphere, 30 °C, 250 mW LED, λ = 530 nm, **8**–**11** identified and quantified by GC–MS.

Although the intramolecular additions of substrates **8** and **10** in the presence of PDI as photocatalyst yielded the corresponding products **9** and **11** only in moderate yields (Scheme 5), they additionally support the proposed photocatalytic mechanism (Scheme 4). Comparison of product formation after 18 h showed that the methoxy substituted product **9** was obtained in approximately double yield compared to **11**. Obviously, the photooxidation of the electron-rich double bond in substrate **8** by electron transfer occured faster than the one in substrate **10**. These results indicate that the initial charge transfer was the rate-limiting step of this photocatalytic process.

The photocatalytic capability of PDI was representatively compared to that of 9-mesityl-10-methylacridinium perchlorate (MesAcr) which was applied by Nicewicz et al. for similar additions [20–21]. After 3 h irradiation at 448 nm by two LEDs (250 mW) in the presence of MesAcr (otherwise identical experimental conditions as those described in Table 1) product **5** was formed in 30% yield, whereas the corresponding reaction with PDI as the photocatalyst yields 49% when PDI is irradiated at 530 nm and 59% when irradiated at 470 nm. These irradiations were performed with the corresponding LEDs and yields were identical with conversions.

Finally, the nucleophilic addition of methanol to **1** using PDI as photocatalyst was representatively executed in two mesoflow reactors, since flow chemistry has significant advantages over batch chemistry, such as easier temperature control, larger surface-to-volume ratio and more efficient photoirradiation. Two setups were used to transfer the reaction to continuous-flow systems. The first mesoflow reactor was equipped with four 250 mW high-power LEDs (λ = 530 nm), a syringe pump, and temperature control to 30 °C. The second one was constructuted for exposure to sunlight and consisted of a PTFE tubing to demonstrate applicability of this photocatalysis without need for electricity. Mesoflow experiments were executed using either sunlight, to give 72% yield over only 1 h, or four high-power LEDs, to give 76% yield over 3 h (Table 2). As control that **1** was not excited directly by sunlight, a sample without PDI was set into sunlight and, as expected, yielded no product.

**Table 2:** Photocatalytic experiments with **1** in flow reactors^a^.

setup	yield of **5** (%)

mesoflow reactor 1^b^	76^c^
mesoflow reactor 2^d^	72^e^
sunlight w/o PDI^f^	0

^a^Reaction conditions: **1** (25 mM), Ph–SH (12.5 mM), PDI (0.5 mM), in CH_2_Cl_2_/MeOH 3:1, argon atmosphere, reactants identified and quantified by GC–MS. ^b^Syringe pump with flow rate of 300 µL/h, 220 min, 30 °C, 4 × 250 mW LED, λ = 530 nm. ^c^Conversion = 88%. ^d^4 mL, rt, sunlight, 17/04/14, Karlsruhe, 11 a.m. until noon. ^e^Conversion = 100%. ^f^1 h, no conversion; 1 month, 51% conversion mainly to benzophenone.

## Conclusion

The photocatalytic complementarity of the two different routes to either the Markovnikov- or anti-Markovnikov-type nucleophilic alcohol addition to styrene derivatives was accomplished by Py and PDI as photoredox catalysts. The regioselectivity was controlled by the type of photoinduced charge transfer that was initiated by the photoexcited catalyst. For the reductive mode towards the Markovnikov orientation, Py was applied as photocatalyst. It was previously elucidated that inefficient back electron transfer required the addition of Et_3_N as electron shuttle that closed the photocatalytic cycle since back electron transfer occurred more efficiently. The photocatalytic process was used also for intramolecular additions. For the oxidative mode towards the anti-Markovnikov-type regioselectivety, PDI was a highly suitable photocatalyst based on its electrochemical and optical properties. Photocatalytic additions of a variety of alcohols to styrene derivatives gave the corresponding products in good to excellent yields. Similar to the reductive mode, the oxidative nucleophilic addition needed the additive Ph–SH as electron and proton shuttle. The proposed photocatalytic electron transfer mechanism was supported by the observation of the PDI radical anion as key intermediate and by comparison of two intramolecular reactions with different electron density. Representative mesoflow reactor experiments revealed that the irradiation times can be significantly shortened and sunlight can be used as a “green” light source. The yields of methanol addition using PDI as photocatalyst were higher than those obtained with MesAcr as literature-known photocatalyst. These results provide a good basis to extend this photocatalytic approach to other nucleophilic additions as synthetically valuable olefin functionalizations, including C–C bond formations.

## Experimental

**Materials and methods.** All chemicals were purchased from Aldrich, ABCR and TCI. GC–MS data were recorded on a Varian GC–MS System (gas-phase chromatograph 431-GC, mass spectrometer 210-MS). Absorption spectra were determined with a Perkin Elmer Lambda 750 UV–vis spectrometer. Fluorescence was measured with a Horiba Scientific FluoroMax 4 spectrofluorometer with step width of 1 nm and an integration time of 0.2 s.

**Photocatalytic experiments with Py.** Irradiations have been executed in a 4 mL cuvette equipped with a magnetic stir bar. The samples were prepared with stem solutions and final concentrations of the substrates (2 mM) and Py (2 mM) in MeCN. The solution was then degassed using the freeze pump thaw method and afterwards irradiated with a 366 nm LED while stirring. Samples have been taken under argon counterflow to prevent oxygen from getting into the reaction mixture.

**Photocatalytic experiments with PDI.** Irradiations have been executed in a 4 mL cuvette equipped with a magnetic stir bar. The samples were prepared with stem solutions and final concentrations of the substrates (25 mM), Ph–SH (12.5 mM) and PDI (0.5 mM) in either CH_2_Cl_2_ or CH_2_Cl_2_/alcohol 3:1 mixtures. The solution was then degassed using the freeze pump thaw method and afterwards irradiated with a 530 nm LED while stirring. Samples have been taken under argon counterflow to prevent oxygen from getting into the reaction mixture.

## Supporting Information

File 1 Spectral data: Cyclic voltammogram of PDI, determination of *E*_00_ of PDI, Stern–Volmer plots of PDI in the presence of substrate **1**, spectroelectrochemistry of PDI, pictures of the mesoflow setups.
