# Supplementary material for: Photocatalytic nucleophilic addition of alcohols to styrenes in Markovnikov and anti-Markovnikov orientation
Source: Beilstein J Org Chem. 2015 Apr 27;11:568–75. doi: 10.3762/bjoc.11.62 (PMC7877480; doi:10.3762/bjoc.11.62)
Supplement: File 1 — Spectral data: Cyclic voltammogram of PDI, determination of E00 of PDI, Stern–Volmer plots of PDI in the presence of substrate 1, spectroelectrochemistry of PDI, pictures of the mesoflow setups. [file Beilstein_J_Org_Chem-11-568-s001.pdf]

Supporting Information

for

Photocatalytic nucleophilic addition of alcohols to styrenes in Markovnikov and anti-Markovnikov orientation

Martin Weiser[1], Sergej Hermann[1], Alexander Penner[1] and Hans-Achim Wagenknecht*[1]

Address: [1]Institute of Organic Chemistry, Karlsruhe Institute of Technology (KIT), Fritz-Haber-Weg 6, 76131 Karlsruhe, Germany

Email: Hans-Achim Wagenknecht - Wagenknecht@kit.edu

* Corresponding author

Spectral data: Cyclic voltammogram of PDI, determination of E_{00} of PDI, Stern–Volmer plots of PDI in the presence of substrate 1, spectroelectrochemistry of PDI, pictures of the mesoflow setups.

Contents

1. Cyclic voltammogram of PDI S2
2. Determination of E_{00} of PDI S3
3. Stern–Volmer plots of PDI in presence of substrate **1** S4
4. Spectroelectrochemistry of PDI S5
5. Setup of mesoflow reactors S6

1. Cyclic voltammogram of PDI

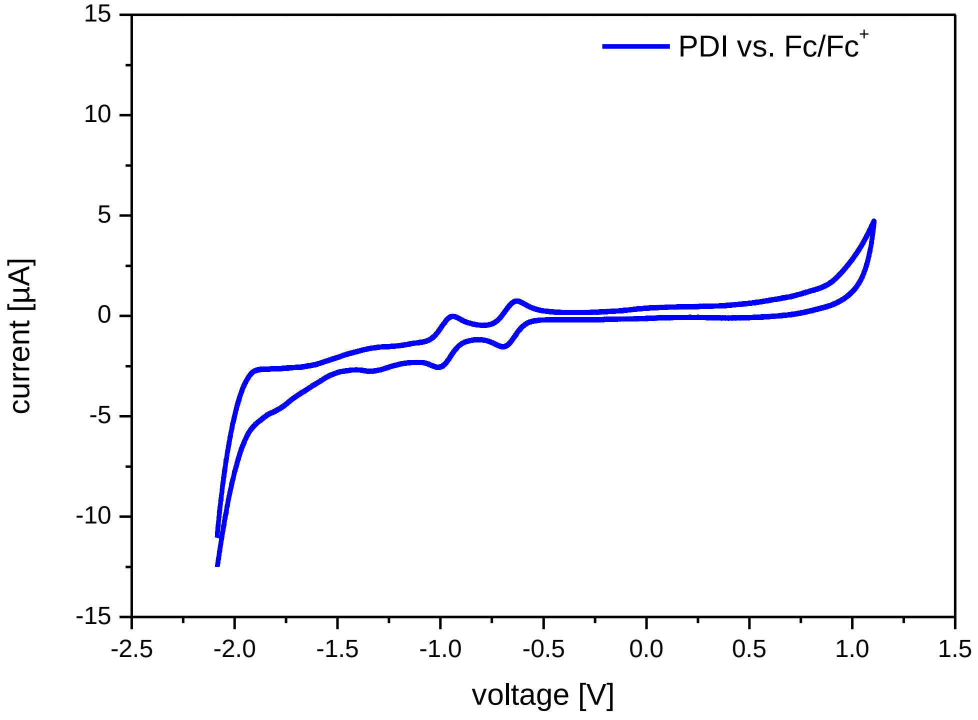

Figure S1: Cyclic voltammogram of PDI (vs Fc/Fc$^+$), 20 mV/s, 0.1 M in TBAHFP in CH_2Cl_2, E_{red}(PDI/PDI$^{•-}$) = −0.66 V (vs Fc/Fc$^+$); E_{red}(PDI/PDI$^{•-}$) = −0.28 V (vs SCE) [1].

2. Determination of E_{00} of PDI

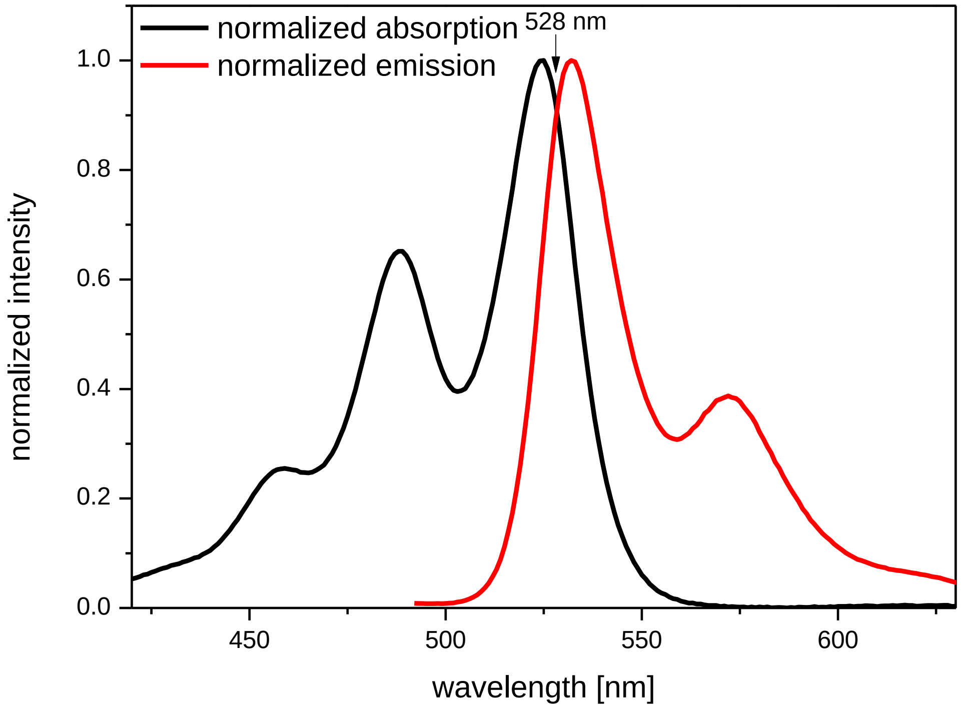

Figure S2: normalized absorption (black) and emission (red) spectra of PDI in CH_2Cl_2. λ_{abs} = 525 nm, λ_{em} = 530 nm, λ_{int} = 528 nm, E_{00} = 2.35 eV.

3. Stern–Volmer plots of PDI in presence of substrate 1

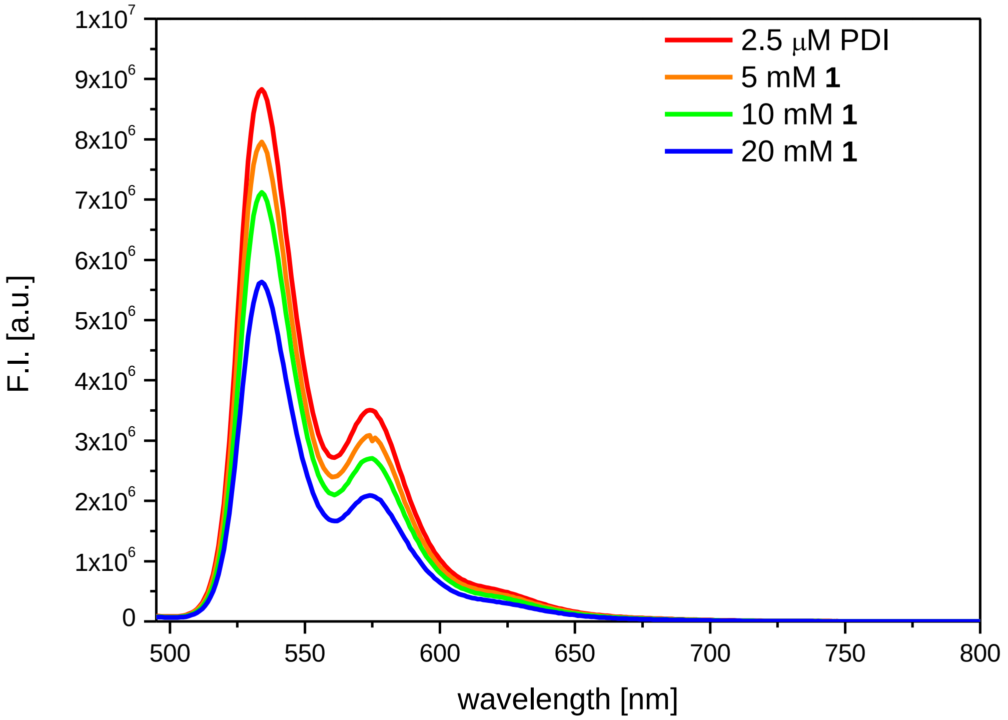

Figure S3: Emission spectra of PDI (2.5 μM) without (red) and with concentrations of 5 (orange), 10 (green) and 20 mM (blue) of **1** in CH_2Cl_2; λ_{exc} = 487 nm.

4. Spectroelectrochemistry of PDI

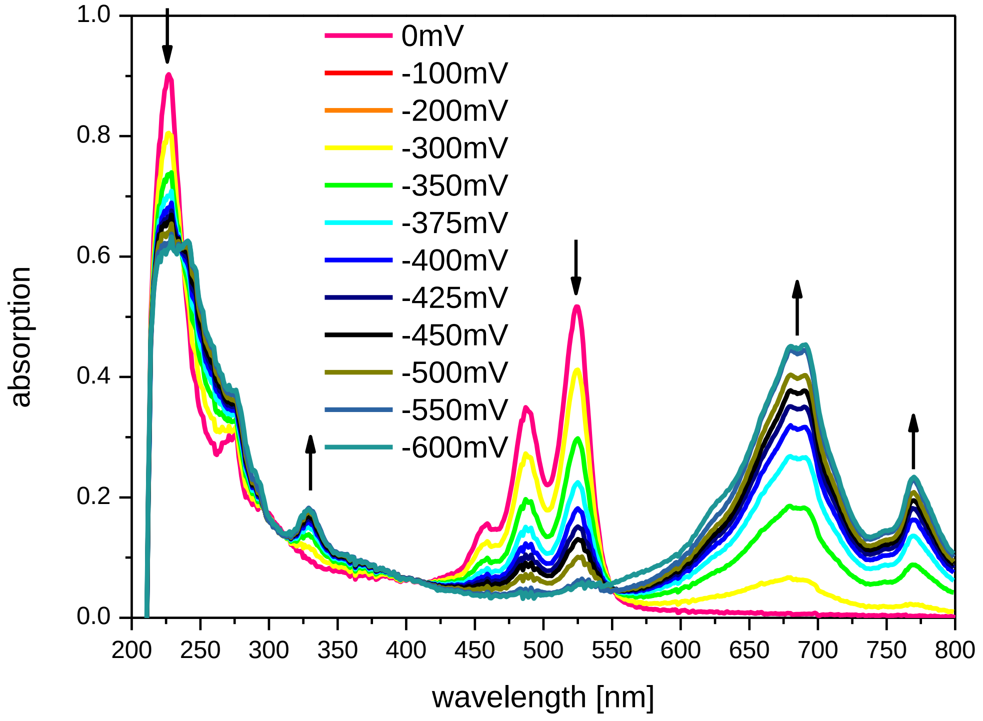

Figure S4: Spectroelectrochemistry of PDI in CH_2Cl_2.

5. Setup of mesoflow reactors

Mesoflow reactor 1 is the commercially available borosilicate glass reactor LTF-VS, purchased from the little things factory (http://www.ltf-gmbh.com/produkte/mr_lab.html) with an inner diameter of 1 mm and dimension of 115 × 60 × 6 mm (L, B, H).

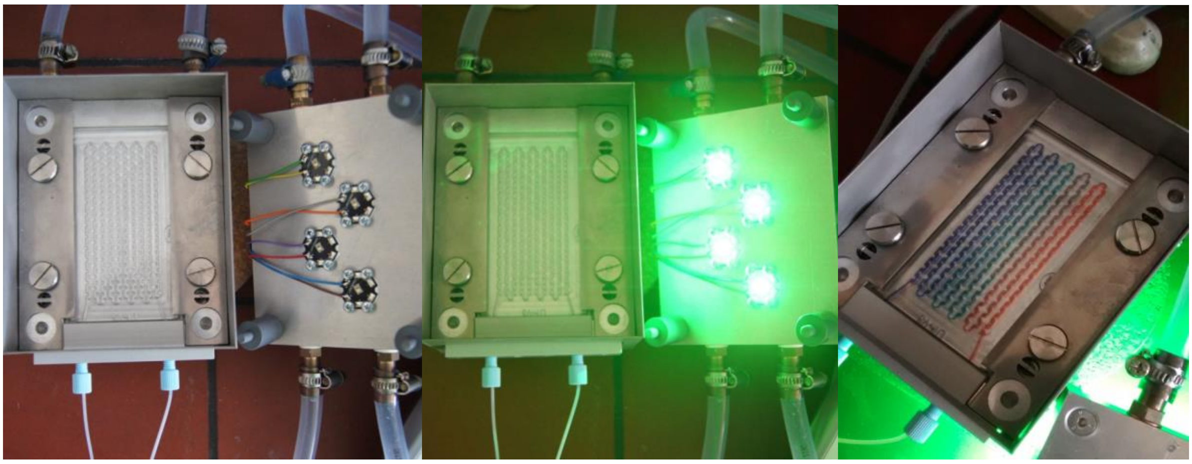

Mesoflow reactor 2 is made of PTFE tubing with an inner diameter of 1.0 mm wrapped around a cooling finger. Pressure was controlled with an argon balloon.

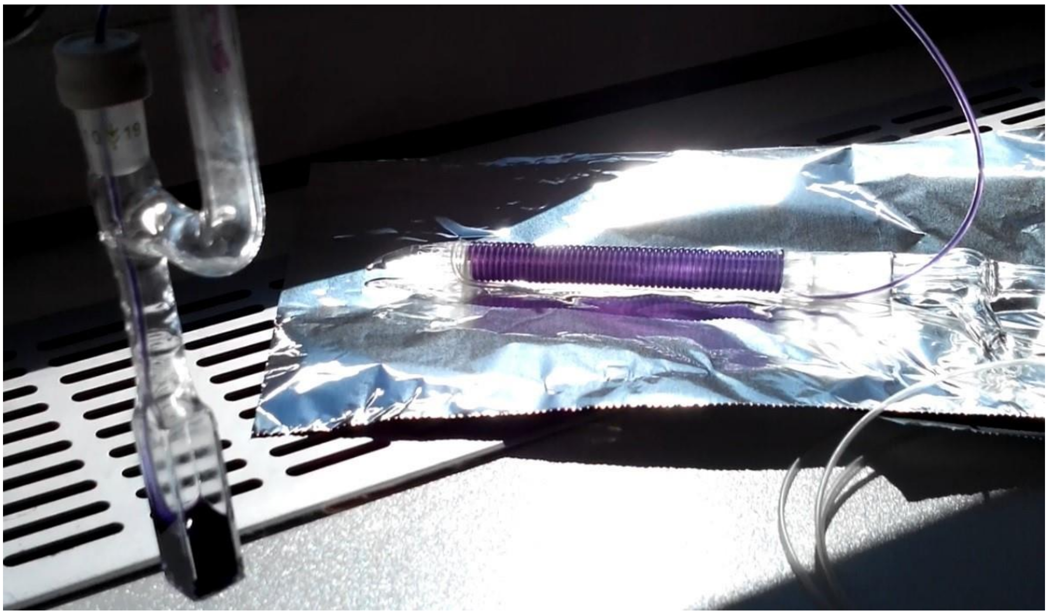

References

1. Pavlishchuk, V. V.; Addison, A. W., *Inorg. Chim. Acta* **2000,** *298*, 97-102.
